# To Parents

**Published:** 1875-12

**Authors:** 


### TO PARENTS.

You surely cannot be ignorant of the fact that you are cheating your children out of the food which gives blood, and muscle, and brains, when you set before them white bread. You must have learned ere this, that the flour is robbed of its best part, when all except the white portion is removed by bolting. Bread made from white flour is simply bread made from starch, and starch is not food. It is not necessary, it is not generally proper, that the substitute should be the coarse harsh meal known as "Graham." The stomach acts upon more finely divided flour with great ease and readiness. But it is vastly important that the whole of the grain should enter into the flour; or that, if anything is sifted out and given to the hens, or used in stiffening linen, it should be the very portion which is now barrelled up and sold as flour.

